# The impact of hearing impairment and noise-induced hearing injury on quality of life in the active-duty military population: challenges to the study of this issue

**DOI:** 10.1186/s40779-016-0082-5

**Published:** 2016-04-12

**Authors:** Hasanat Alamgir, Caryn A. Turner, Nicole J. Wong, Sharon P. Cooper, Jose A. Betancourt, James Henry, Andrew J. Senchak, Tanisha L. Hammill, Mark D. Packer

**Affiliations:** Deptartment of Health Policy and Management, School of Health Sciences and Practice, New York Medical College, Valhalla, NY 10595 USA; School of Public Health, The University of Texas Health Science Center at Houston, 7411 John Smith Drive, Suite 1100, San Antonio, TX 78229 USA; National Center for Rehabilitative Auditory Research, 3710 SW US Veteran Hospital Road, Portland, OR 97239 USA; Walter Reed National Military Medical Center, 8901 Wisconsin Ave, Bethesda, MD 20814 USA; Department of Defense Hearing Center of Excellence, 59MDW/SG02O, 2200 Bergquist Drive, Suite 1, JBSA Lackland, TX 78236 USA

**Keywords:** Quality of life (QOL), Hearing impairment, Noise-induced hearing injury, Hearing loss, Tinnitus, Hearing impairment and noise-induced hearing injury (HINIHI), Military, Service members

## Abstract

The objectives of this research were to 1) summarize the available evidence on the impact of hearing loss on quality of life (QOL) among U.S. active-duty service members, 2) describe the QOL instruments that have been used to quantify the impact of hearing loss on quality of life, 3) examine national population-level secondary databases and report on their utility for studying the impact of hearing loss on QOL among active-duty service members, and 4) provide recommendations for future studies that seek to quantify the impact of hearing loss in this population. There is a lack of literature that addresses the intersection of hearing impairment, the military population, and quality of life measures. For audiological research, U.S. military personnel offer a unique research population, as they are exposed to noise levels and blast environments that are highly unusual in civilian work settings and can serve as a model population for studying the impact on QOL associated with these conditions. Our team recommends conducting a study on the active-duty service member population using a measurement instrument suitable for determining decreases in QOL specifically due to hearing loss.

## Background

Auditory system disabilities are some of the leading diagnoses among the veterans of Operations Enduring Freedom and Iraqi Freedom, with tinnitus and hearing loss being the two most prevalent service-connected (SC) disabilities among veterans, both overall and among the new SC disability awards given during fiscal year (FY) 2013 [1]. The economic burden of SC auditory system disabilities, which include tinnitus, hearing loss and impaired hearing, is immense for the Veterans Health Administration (VA), as evidenced by the more than 2.1 million veterans in FY 2013 who received SC auditory system disability compensation [1]. These SC disability awards create an economic burden from the VA’s perspective, and auditory system disabilities have a great impact on the quality of life (QOL) of affected individuals while on active duty or post-service [2].

Recommendations from the 2005 Institute of Medicine report on Noise and Military Service included a call for studies to estimate the incidence, prevalence, and severity of noise-induced hearing loss (NIHL) and tinnitus [3]. Despite the high number of SC cases involving these auditory conditions, their impact on QOL among U.S. active-duty service members (SMs) has not been studied. The published literature has consistently indicated an association between auditory disorders and decreased QOL measures in the civilian population, revealing detrimental effects on social, psychological, and cognitive functions [4, 5]. Auditory disorders impact many dimensions of health and wellbeing. Therefore, understanding how QOL is reduced by auditory dysfunction is a vital component in determining the comprehensive burden that goes beyond just the economic impact. However, there are numerous challenges to determining the impact on the QOL of active-duty SMs and veterans.

Epidemiological estimates on Americans living with auditory disorders vary based on the definition used and the population sampled, but studies agree that auditory disorders are a growing problem in the U.S., especially with a rapidly aging population [6, 7]. A survey conducted by Kochkin [7] in 2005 found that the number of hearing-impaired people in the U.S. increased from 31.5 million to 35 million, a 9 % increase, during a period when the population grew by only 4.5 %. The report projected that the numbers of Americans with hearing loss will increase to 40 million by 2025 and to 53 million by 2050 [7]. However, in 2011, Lin et. al. used National Health and Nutritional Examination Survey (NHANES) results to estimate the total number of Americans living with hearing loss and concluded that approximately 30 million Americans (12.7 % of the population) aged 12 years and older have bilateral hearing loss. When unilateral hearing loss is added, the estimate balloons to 48.1 million Americans — 20.3 % of the population [6]. The prevalence of hearing loss is higher among men, those with lower educational attainment and lower income, and those in certain occupations and industries [8, 9]. In audiology research, U.S. Military personnel offer a unique research population, as they are exposed to noise levels and blast environments that are highly unusual in civilian work-settings, and they can serve as a model population for studying the impact of hearing loss on QOL. According to one study, between 2007 and 2010, reported cases of sensorineural hearing loss and a significant threshold shift increased from 11 % to 23 % among the active-duty population [10]. The VA has reported a steady increase in auditory system disabilities since at least 1999, including a 12 % increase in disability recipients between FY 2012 and 2013 [1, 11–13].

One of stated objectives of the Department of Defense Epidemiology and Economic Burden of Hearing Loss Study (DEEBoHLS), which is led by The University of Texas School of Public Health in conjunction with the Department of Defense Hearing Center of Excellence, is to comprehensively examine the economic losses and other burdens associated with auditory system disabilities [14]. Throughout the course of this ongoing study, the team has conducted a thorough and extensive review of existing studies on this topic and has noted a critical gap in the available evidence on the impact of auditory system disabilities on QOL in the active-duty military population. This report summarizes the findings from this review, outlines the challenges to studying this gap, and offers recommendations for future studies.

The terminology used in this commentary includes “hearing impairment and noise-induced hearing injury” (HINIHI), which is based on our review of the peer-reviewed literature and congressionally mandated reports [14]. Using this definition allows for the inclusion of acute acoustic trauma that occurs due to a single exposure from a sudden eruption of sound, hearing loss as a result of a more insidious cause from continuous or intermittent noise exposure that often develops over time, and tinnitus. However, when referencing specific articles or databases, we will maintain the terminology used that is specific to that article or database.

## Why is this important?

Published studies include evidence on the social, psychological, cognitive, and health effects of HINIHI in the civilian population. HINIHI results in distorted or incomplete communication leading to isolation and withdrawal and subsequently results in lower sensory input. The feeling of a constricted lifestyle negatively impacts the psychosocial well-being of people with HINIHI. HINIHI has been associated with increased mortality (e.g., HINIHI can increase the risk of accident and injury) and can serve as an indicator of neurological decline in older populations [15]. In personal relationships, HINIHI affects the ability to communicate effectively, which can lead to frustration, anger, and antagonism between partners [5]. Even marginal HINIHI can negatively impact an individual’s sense of independence and well-being. If left untreated, HINIHI can lead to decreased cognitive function, lower quality of life, and reduced functional capacity to conduct daily tasks [5]. For those who are still in the workforce, uncorrected HINIHI can also have a negative impact on overall job effectiveness, opportunities for promotion and perhaps lifelong earning power [16]. Many of these findings may apply to the active-duty military and veteran populations as well; however, HINIHI may go under-reported or even un-noticed in younger SMs and veterans. The military population is different when compared to the civilian population with respect to their demographic composition (primarily younger and male), workplace characteristics (very high physical and mental demands, high degree of alertness required, risk of serious injury during combat or training, high degree of loud noise exposure), and organizational policies.

Numerous studies have reported a high incidence of depression, suicide, and post-traumatic stress disorder (PTSD) among the active-duty population [17]. It is possible that undiagnosed and untreated HINIHI may be one of the underlying triggers in some of these severe health outcomes. A study by Smith et al. [18] showed that 8.7 % of current service members have PTSD, and other studies have estimated that as many as 15 % of Vietnam veterans have PTSD [19], as well as 10 % of Gulf War veterans [20]. Psychological disorders that are paired with a hearing impairment (HI), such as tinnitus, have the ability to exacerbate each other [21, 22]. PTSD combined with HINIHI can cause a person to react based on an inaccurate analysis of sensory input from their surrounding area [23]. Higher risks of anxiety, depression, and PTSD coupled with HINIHI can have a compounded impact on QOL among the military population compared to the effects on the civilian population [23]. QOL measures usually incorporate mental, physical, and social aspects to provide a complete picture of an individual’s health and well-being. This research team was not able to identify a single study that reported on the impact of HINIHI on QOL among the active-duty U.S. military population.

## Literature retrieval methods

The study team performed a broad literature review, casting a wide net to gather all relevant hearing- and military-related articles from a variety of domains. The literature review was updated throughout the study period on a monthly basis. However, although it is comprehensive, this literature review is not intended to be a systematic review.

The literature search regarding hearing loss in the military was carried out using PubMed and Ovid Medline for articles published between 1990 and September 2015 using an electronic key-word search complemented by manual searching. Search terms for the literature review included “hearing”, “hearing disorders”, “hearing loss”, “hearing tests”, “hearing aids”, “audiology”, “hearing injury”, “hearing trauma”, “hearing impairment”, “hearing sensitivity”, “audiological”, “auditory injury”, “auditory trauma”, “noise induced”, “dual sensory”, “hearing threshold”, “hearing conservation”, “hearing deficiency”, “deaf”, “tympanic membrane”, “tinnitus”, “military”, “military personnel”, “military medicine”, “military facilities”, “military science”, “army”, “marine”, “soldier”, “air force”, “navy”, “national guard”, “deployment”, “combat”, “active component”, “active duty”, “armed forces”, “department of defense”, “veterans’ health”, “veterans”, “united states department of veteran affairs”, “veterans”, “disability claims”, “veterans hospitals” and “quality of life”. In addition, a web search was also performed for government reports and reports from individual organizations that were related to hearing impairment and quality of life.

A total of 300 articles and reports were identified by the literature search (236 journal articles, 16 reviews articles, 4 editorials, 41 government reports/other institutional reports, 2 books and 1 dissertation abstract). Of these, Table 1 summarizes the 6 studies that were identified as reporting on the relationship of interest to our study.

**Table 1:** Summary of studies investigating the relationship between quality of life and hearing loss measures among the veteran military population

Study	QOL measurement	Population	Findings/conclusions
Mulrow et al. (1990) [15]	Hearing Handicap Inventory for the Elderly (HHIE), Quantified Denver Scale of Communication Function (QDS), Short Portable Mental Status Questionnaire (SPMSQ), Geriatric Depression Scale (GDS), Self-Evaluation of Life Function (SELF)	Older adult veterans	Hearing loss is associated with important adverse effects on the quality of life of elderly persons, effects which are reversible with hearing aids.
Quality-of-life changes and hearing impairment randomized trial.			
Mulrow, Tuley, and Aguilar, (1992) [24]	HHIE, QDS, GDS, SPMSQ	Hearing impaired veterans	All QOL areas improved significantly from baseline to 4 months. Social and emotional HHIE, communication QDS, and depression GDS benefits were sustained at 8-12 months, but cognitive changes reverted to baseline at 12 months SPMSQ.
Sustained benefits of hearing aids			
Hawkins et al. (2012) [30]	Veterans RAND 12-item	Data from Health Update Survey –10 % of 5,515 eligible adults with AARP Medicare Supplement Plan responded	Hearing impairment was strongly associated with a lower quality of life from both a physical and mental health standpoint.
The prevalence of hearing impairment and its burden on the quality of life among adults with Medicare Supplement Insurance			
Tambs (2004) [37]	One-page hearing questionnaire which included symptoms checklist-25, four tapping symptoms of anxiety and 6 tapping depression. HUNT Q1 and Q2	50,398 subjects, ages 20-101, Norway, included those who served in military service	Hearing loss is associated with substantially reduced mental health ratings among some young and middle-aged persons but usually does not significantly affect mental health among older persons.
Moderate Effects of Hearing Loss on Mental Health and Subjective Well-Being: Results from the Nord-Trondelag Hearing Loss Study			
Abrams, Chisolm, McArdle (2002) [28]	Medical Outcomes Study 36-item Short-Form – modified for Veteran population (SF-36 V)	Veterans	Of the two arms in the study, the arm that was given both the hearing aid and audiological rehabilitation interventions saved $28.09 per quality-adjusted life year (QALY) compared to hearing aid intervention only arm.
A cost-utility analysis of adult group audiologic rehabilitation: Are the benefits worth the cost?			
Yueh et al. (2010) [26]	RAND-36 (formerly called SF-36 V)	Veterans	No statistically significant differences in RAND-36 measurements were found.
Long‐Term Effectiveness of Screening for Hearing Loss: The Screening for Auditory Impairment—Which Hearing Assessment Test (SAI‐WHAT) Randomized Trial			

## Summary of previous studies and the QOL instruments used

Several studies have considered the association between QOL and HINIHI in veteran and civilian populations. A study by Mulrow et al. [24], considered the impact of HI on QOL specifically among veterans. The study determined that HI was associated with adverse effects on the QOL of older adults and, importantly, that these effects were reversible with hearing aids [24]. The few studies that evaluated the impact of HI on QOL among veterans primarily considered changes in QOL after an intervention, typically hearing aid use [15, 24–26].

The majority of studies investigating the relationship between QOL measures, veteran status, and HINIHI used the SF-36, which is a validated survey that contains a standardized set of 36 questions developed by the Medical Outcomes Study (MOS) in America [27]. Two summary scores are derived from the SF-36 results: 1) the Physical Component Score (PCS), which highlights the individual’s physical functioning, role-physical, bodily pain, and general health; 2) the Mental Component Score (MCS), which emphasizes vitality, social functioning, and role-emotional. Abrams et al. [28] modified the SF-36 for the veteran population and named it the SF-36 V. This instrument has been modified further and given several names, including the RAND-36, RAND-12 (a modified, 12-question version of the RAND-36), Veterans RAND-12 item, VR-36 and the VR-12 [29–31]. Researchers investigating QOL for specific disabilities have shown that patients score lower on specific SF-36 items because of functional limitations [32]. Magnusson et al. [32] have also modified the SF-36 to specifically highlight headaches. Their studies found significantly different scores between their modified version of the SF-36 and the standard SF-36.

Feeney et al. [33] used the Health Utilities Index Mark 3 (HUI3) to measure QOL in their 2012 study on the use of hearing, mobility, and pain to predict mortality. This questionnaire measures eight attributes of health-related quality of life (HRQL), including vision, hearing, speech, ambulation, dexterity, cognition, emotion, pain, and discomfort on a scale of five or six for each attribute [33]. The authors found a significant association between QOL and the ability to predict mortality in groups with reported HI or pain [33].

Mulrow et al. [15] used a combination of the Hearing Handicap Inventory for the Elderly (HHIE), Quantified Denver Scale of Communication Function (QDS), Short Portable Mental Status Questionnaire (SPMSQ), Geriatric Depression Scale (GDS), and the Self-Evaluation of Life Function (SELF). The HHIE and QDS are specific for hearing loss while the SPMSQ, GDS, and SELF are all generic tools to assess QOL. None of these QOL instruments were made or tailored specifically for active-duty or veteran populations [15].

Veterans Affairs Canada (VAC) takes QOL into consideration when determining the total percentage of disability for SMs [34]. The QOL measure used by the VAC is a general instrument and the same criterion is used for all disabilities. Impacts on QOL are rated as level 1, 2, or 3 (mild, moderate, or extreme, respectively), each level is dependent on the service member’s ability to participate in the following areas: domestic chores, vehicle transport/travel, recreational activities, and inter-personal relationships. It also assesses how they are affected by the specific SC disability [34]. The QOL rating is based on the medical information provided to the VAC during the application process; as a result, there is no official QOL measurement that service members fill out. Instead, the rating is based completely on the subjective judgment of the VAC worker [34].

There are QOL measurement instruments that do not appear to have been used as frequently in HINIHI studies; they include the HHIE, QDS, SPMSQ, GDS, SELF, Mini-Mental State Examination (MMSE), Psychological General Well-being Index, International Outcome Inventory-Cochlear implant (IOI-CI), and the Nord-Trondelag Health Study (HUNT) Q1 and Q2 [15, 24, 35–37]. However, none of these measurements were developed for or applied to active-duty populations. This suggests a gap in our knowledge about suitable hearing health-related QOL measurement tools and the impacts of HINIHI on QOL in military populations.

## Are there databases available to study hearing impairment and QOL measures in active-duty service members?

The following databases (Table 2) were explored to determine the availability of secondary national-level datasets to study the impact that HINIHI has on QOL in active-duty SMs: NHANES, National Health Interview Survey-Occupational Health Supplement (NHIS-OHS), Behavioral Risk Factor Surveillance System (BRFSS), National Health Interview Survey (NHIS), and the Health and Retirement Study (HRS).

**Table 2:** Summary of databases explored and the relevancy to the interests of this investigation

Database name	Organization	Remarks	Relevance to investigation interests
National Health and Nutrition Examination Survey (NHANES)	CDC	Section on “Hearing/audiometry (AUQ) for 1 year and over, 1999-2012 with component or lab tests conducted on original sample description. In 2013-2014, there was no component/lab test conducted.	Yes
National Health Interview Survey Occupational Health Supplement (NHIS-OHS)	CDC	Does not contain any audiometric topics.	No
Behavioral Risk Factor Surveillance System (BRFSS)	National Center for Chronic	Option to respond ‘hearing problem’ as the major impairment that limits activities 1998-2002, 2013; Veteran’s Status included, 2003-2008, 2010-2012	Yes
	Disease Prevention and Health Promotion (NCCDPHP)		
National Health Survey	CDC	Questions by family and individuals on hearing can derive some information about associations with the military, data available from 1997 onward. QOL is a supplemental (optional) component of the survey, available 2010-2012	Yes
Health and Retirement Study (HRS)	University of Michigan	Data from 1992-2014 on hearing status, employment, and QOL measures	Yes

The NHIS-OHS is a survey on current workplace exposures and occupation-related health conditions that does not contain any topics related to noise exposure or HINIHI [38]. The BRFSS is a telephone survey that gathers information related to health risk behaviors, chronic health conditions, and the use of available preventative services. The BRFSS varies from state to state in regard to the information gathered in the surveys throughout the years, but it does not gather information pertaining to QOL or HINIHI [39].

The NHANES is a battery of cross-sectional studies that assess the physical health and nutritional status of non-institutionalized adults and children. The NHANES standard survey contains some questions that are related to QOL indicators, which are given to all participants, and includes a physical examination and lab test component. The audiometric component is only given to those in the targeted age range, which varies from survey to survey. The audiometric component is not given during some years and the target age ranges have varied widely, from 6-11 years in 1963-1965, 20-69 years in 1999-2004, and 12-19 and 70+ years old in 2005-2006 and 2009-2010 [40]. However, the data do not distinguish between active-duty and veteran populations. As of 2011, the variables on military service are very broad and only ascertain if a participant ever served in the military and, more recently, if their service was in a foreign country [41, 42]. Additionally, the NHANES survey does not distinguish between active-duty and veteran participants, which presents a challenge in collecting data on active SMs.

The NHIS is a cross-sectional, household interview survey for the civilian non-institutionalized population that has been conducted continuously since 1957 [43]. The NHIS category related to HINIHI is “Hearing Problem”. In both child and adult surveys, participants were asked whether a doctor or other health professional has told them they had the condition in question (hearing impairment, for example). If the participant indicated having a HI, they would then be asked about past or present hearing aid use [44]. The data exclude active-duty service members due to the study population being defined as “civilian, non-institutionalized.” There are questions in the socio-demographic section that ask if adult respondents have ever served on active-duty in the U.S. Armed Forces, Military Reserve Forces, or in the National Guard. These questions can only ascertain veteran status due to the exclusions previously outlined, which means that the NHIS is not useful in addressing the current study’s question [43].

The HRS is a longitudinal study of health, retirement, and aging sponsored by the National Institute on Aging [45]. The University of Michigan has surveyed a representative sample of over 26,000 American civilians over the age of 50 every two years. Data are available from 1992 to 2014 on the demographics, hearing status, health care utilization and costs, functional limitations (ADLs/IADLs), cognition, income, employment status and employment history of the respondents. Because this study targets a population that is much older than the relatively young military population, it holds little merit for the current study.

From our review of these available national-level databases, none contain the data necessary to assess the association of HINIHI and QOL in the U.S. military population. Therefore, we make specific recommendations below to help fill this research gap.

## Conclusions: Future direction and recommendations

Given the lack of universal agreement on the definition of QOL, there is no consensus regarding the best method for determining QOL [25]. Issues have been raised about using generic instruments versus disease-specific instruments. Disease-specific instruments are better at determining the effects that a specific health condition, illness, or disease has on the various dimensions of QOL, as well as being sensitive enough to detect changes brought on by an intervention. This helps in conducting studies comparing the effectiveness of interventions for the same health condition and in detecting longitudinal changes due to an intervention. However, disease-specific instruments are unable to compare the QOL scores across disorders and interventions [25]. Examples of disease-specific instruments include the Abbreviated Profile of Hearing Aid Benefit (APHAB) and the HHIE [15, 24, 25]. Generic instruments primarily focus on an individual’s self-perceived overall health status [25]. A major issue in selecting a suitable instrument for assessing QOL is the variety of attributes assigned to the physical, social, and mental health domains; the lack of consistency in the terminology used for the domains; and the QOL attributes used in the various measurement instruments [25]. There can also be differences in an instrument’s focus for use in obtaining “profile” or “utility” (preference-based) measures. Profiles attempt to measure all of the important aspects of QOL by assessing and scoring each domain separately and then compiling the scores into a summary measure. Most generic QOL instruments are classified as profiles, such as the SF-36 and SELF [25]. Utility refers to the preference an individual expresses for a particular health state [25]. For example, a utility measure may ask an individual if he/she would rather live longer and risk acquiring a particular health condition or live a shorter life without the risk of acquiring that particular health condition. Utility measures have been criticized because decisions about risk-taking and time trade-offs cause cognitive biases in participants, and the analog scale used is not a true preference-based measure [25]. The HUI3 is a utility measure, but it has primarily been utilized in cost analysis studies for cochlear implants [25].

Studies focused on civilian populations have consistently detected an association between HINIHI and decreased QOL measures. Our findings suggest that the SF-36 was the most frequently cited survey instrument for assessing QOL in both civilian and veteran populations in relation to HINIHI and several other health conditions. However, as outlined previously, the utility of the results from the SF-36 have been mixed when the survey has been altered to specifically address certain health conditions [28–30, 32]. We propose to use the original version of the SF-36 so that the results can be compared to other studies and health conditions. In addition to the SF-36, we propose that a HINIHI-specific QOL instrument be used to detect hearing-specific dimensions with greater sensitivity and specificity. Most of these QOL instruments are simple, quick to administer, and fairly easy to understand, and their validity and reliability have been well established by previous research [15, 24, 25].

Among the secondary databases evaluated, there was a lack of information that covered the intersection of HINIHI and QOL measures in active-duty military populations. These databases may, at best, be used to investigate the association between hearing impairment and some QOL measures (BRFSS, HRS) or hearing impairment and its association with the veteran status of a population (NHIS). None of these secondary databases contains QOL information that pertains to active-duty SMs in relation to HINIHI.

Given the high prevalence and burden of HINIHI in military populations, it is imperative to conduct a study that captures the impact of these conditions on the QOL of active-duty SMs. It is our recommendation that future studies collect prospective and primary data from randomly sampled active-duty service members using 1) a generic QOL instrument, such as the SF-36, and 2) a specific QOL instrument for assessing HINIHI. To ensure that the results are generalizable, this study population should be stratified so that major demographic (age, sex, race/ethnicity) and occupational characteristics (e.g., branch, military occupational specialty, deployments) are appropriately represented. We also recommend that future studies determining and reporting on the burden of any health condition and/or evaluating interventional outcomes in active-duty service members incorporate appropriate QOL measures.

## Abbreviations

ADL: activities of daily living
APHAB: abbreviated profile of hearing aid benefit
BRFSS: behavioral risk factor surveillance system
DEEBoHLS: Department of Defense Epidemiology and Economic Burden of Hearing Loss Study
FY: fiscal year
GDS: geriatric depression scale
HHIE: hearing handicap inventory for the elderly
HI: hearing impairment
HINIHI: hearing impairment and noise-induced hearing injury
HRQL: health-related quality of life
HRS: health and retirement study
HUI3: health utilities index mark 3
HUNT: Nord-Trondelag health study
IADL: instrumental activities of daily living
IOI-CI: International Outcome Inventory- Cochlear Implant
MCS: mental component score
MMSE: mini-mental state examination
MOS: medical outcomes study
NHANES: National Health and Nutritional Examination Survey
NHIS: National Health Interview Survey
NHIS-OHS: National Health Interview Survey- Occupational Health Supplement
NIHL: noise-induced hearing loss
PCS: physical component score
PTSD: post-traumatic stress disorder
QDS: quantified Denver scale of communication function
QOL: quality of life
SC: service-connected
SELF: self-evaluation of life function
SF: standard form
SM: service member
SPMSQ: short portable mental status questionnaire
VA: veterans health administration
VAC: veterans affairs Canada
VR: veterans RAND
